# Role of 2‒^13^C Isotopic Glyphosate Adsorption on Silver Nanoparticles Based on Ninhydrin Reaction: A Study Based on Surface—Enhanced Raman Spectroscopy

**DOI:** 10.3390/nano10122539

**Published:** 2020-12-17

**Authors:** Meng-Lei Xu, Yu Gao, Jing Jin, Jin-Feng Xiong, Xiao Xia Han, Bing Zhao

**Affiliations:** 1 College of Food Science and Engineering, Jilin University, Changchun 130062, China; xuml13@mails.jlu.edu.cn; 2 State Key Laboratory of Supramolecular Structure and Materials, Jilin University, Changchun 130012, China; jinjing16@mails.jlu.edu.cn; 3 College of Plant Protection, Jilin Agricultural University, Changchun 130118, China; gaoy1101@163.com; 4 Changchun Institute of Biological Products, Changchun 130012, China; xjfzhuang@163.com

**Keywords:** glyphosate, isotopic Raman shifts, SERS, ninhydrin reaction

## Abstract

Glyphosate is one of the most commonly used and non-selective herbicides in agriculture, which may directly pollute the environment and threaten human health. A simple and effective approach to its detection is thus quite necessary. Surface-enhanced Raman scattering (SERS) spectroscopy was shown to be a very effective method to approach the problem. However, sensitivity in SERS experiments is quite low, caused by different orientation/conformation of the adsorbed molecules on the metal surface, which limit its detection by using SERS. In this paper, 2‒^13^C‒glyphosate (hereafter: 13–GLP) was designed as a model molecule for theoretical and experimental studies of the molecule structure. Vibrational modes were assigned based on the modeling results obtained at the B3LYP/6-311++G** level by density functional theory (DFT) calculations, which were performed to predict the FT‒IR and Raman spectra. Band downshifts were caused by ^13^C atom isotopic substitution with mass changed. Moreover, SERS spectra of 13–GLP by combining ninhydrin reaction on Ag NPs were obtained. Isotopic Raman shifts are helpful in identifying the components of each Raman band through vibrations across the molecular system. They are coupled by probe molecules and thus bind to the substrates, indirectly offering the opportunity to promote interactions with Ag NPs and reduce the complex equilibrium between different orientation/conformation of glyphosate molecules on the metal surface.

## 1. Introduction

Glyphosate (C_3_H_8_NO_5_P, CAS: 1071‒83‒6) is one of the most commonly used and non-selective herbicides in agriculture, which may directly pollute the environment and threaten human health. Glyphosate was first synthesized by the Monsanto company and marketed under the name “Roundup”, and then became well known as a common herbicide to control weeds. Based on its chemical properties, glyphosate is expected to be adsorbed by the soil; due to its widespread use, it directly pollutes the environment and contaminate foods. The detection of glyphosate residues water, food, and feed stuff is an essential step in regulating and monitoring the level. The majority of these methods are based on either gas chromatography (GC) or high-performance liquid chromatograph (HPLC) coupled to a variety of detectors, mainly mass spectrometric detectors. Most of the methods are highly sensitive, however, in most cases tedious since they include numerous steps for the purification and derivatization of the compound.

Surface-enhanced Raman scattering (SERS) has been widely used as a powerful tool for ultrasensitive chemical analysis, as this technique is sensitive enough to detect glyphosate molecule [1]. Taking glyphosate as an example, although numerous efforts have been made, disagreements are still unresolved regarding the vibration assignments, and a complex equilibrium between different orientation/conformation of glyphosate molecules on the metal surface further prevents an explicit understanding of the glyphosate-detection process at a molecular level by SERS. We reported a simple and sensitive method for the determination of glyphosate by combining ninhydrin reaction and SERS spectroscopy [2]. The product (purple color dye, PD) of the ninhydrin reaction is found to SERS-active and directly correlate with the glyphosate concentration. However, vibration assignments and absorption were not completely clear.

The SERS detection method based on isotope labeling can achieve the purpose of defining vibration mode precisely. Isotope labeling has been reported in the detection of glyphosate residues and its transformation products based on NMR, MS, HPLC, GC, and GC‒MS [3,4]. The SERS detection method based on isotope labeling has its own advantages. The vibration frequency of different isotopes is different, and there will be great differences. In terms of the attribution of characteristic peaks, the molecular vibration of isotope labeling was reported partly, however, the theoretical and practical spectral differences still need to be compared with DFT calculation with clearly defined assignments [5].

In this paper, in order to make more clearly defined assignments to each atom, we determined 2‒^13^C‒glyphosate (13–GLP) as a model molecule for the theoretical and experimental studies of the molecule structure. Isotopic effects on Raman/FT–IR vibrational frequency and intensity due to the change of reduced masses and the vibrational coupling were verified by density functional theory (DFT) simulations and its corresponding isotopic SERS measurements. Density functional theory (DFT) calculations were performed to predict the Fourier Transform Infrared (FT–IR) and Raman spectra for the molecule. In order to reduce the complex equilibrium between different orientation/conformation of glyphosate molecules on the metal surface, a glyphosate detection method coupled by probe molecules with accurate vibration assignments thus bind to the substrates indirectly offered the opportunity to promote interactions with Ag NPs.

## 2. Materials and Methods

### 2.1. Materials

2‒^13^C‒glyphosate (99%, CAS: 287399-31-9, hereafter: 13–GLP), glyphosate (99%, CAS: 1071-83-6, hereafter: 12–GLP), potassium bromide (KBr, powder, 99.999%, CAS: 7758-02-3), and silver nitrate (AgNO_3_, 99.8%, CAS: 7761-88-8) were purchased from Sigma-Aldrich Chemical Co. (Shanghai) (Shanghai, China). Ninhydrin (2,2–dihydroxyindane–1,3–dione, C_9_H_6_O_4_, CAS: 485-47-2, 98%) were obtained from Shanghai Aladdin Bio-Chem Technology Co., LTD (Shanghai, China). Sodium citrate (Na_3_C_6_H_5_O_7_·2H_2_O, CAS: 6132-04-3, 99%), sodium molybdate (Na_2_MoO_4_, CAS: 7631-95-0, 99%), and all other chemicals were analytical-grade reagents and were purchased from Beijing Chemical Reagent Factory (Beijing, China) and used without further purification. Ultrapure water (18.25 MΩ) was used throughout the experiments.

### 2.2. Instruments

Raman spectra were recorded on a Jobin Yvon/HORIBA LabRam ARAMIS Raman spectrometer (Tokyo, Japan) equipped with an integral BX 41 confocal microscope (Tokyo, Japan). Radiation from an air‒cooled internal HeNe laser (632.8 nm) and an external cavity diode laser (785 nm) were used as the excitation source. The FT‒IR spectra of ninhydrin were recorded as KBr disks at room temperature by a Bruker IFS‒66V FT‒IR spectrometer (Karlsruhe, Germany), equipped with a DTGS detector (Karlsruhe, Germany) at a resolution of 4 cm^−1^. 

### 2.3. Preparation of Silver Nanoparticles

Ag NPs were prepared according to the Lee and Meisel method, starting from silver nitrate (36 mg AgNO_3_ added into 200 mL H_2_O) and using sodium citrate (1% ω/V, 4 mL) as reducing agent [6]. After heating for 40 min at 85 °C, a grey‒green colloid formed and was naturally cooled at room temperature.

### 2.4. Preparation of Solution

Na_2_MoO_4_ solution (5%, ω/V) was prepared by dissolving 5.00 g Na_2_MoO_4_ in 100–mL H_2_O. **Ninhydrin working reagent**: ninhydrin solution (5%, ω/V) + water + acetate buffer (0.4 mol·L^−1^, pH 5.5) (2:1:1, V/V/V). Tap water was collected without further clean-up for real sample determination.

### 2.5. Preparation of Purple Color Dye (PD) Product

Ninhydrin working reagent + 5% Na_2_MoO_4_ + 12–GLP/13–GLP solution (1:1:1, V/V). The mixed working solutions were heated in boiling water for 30 min; 10.00 µL of the producing solutions were mixed with 10 µL Ag colloid for SERS measurements [2].

### 2.6. Theoretical Method

All geometries were optimized using the B3LYP exchange-correlation functional, which is a hybrid of Becke’s three-parameter exchange, and the Lee-Yang-Parr correlation functionals [7,8,9]. The 6-311++G(d,p) basis set was used for the H, C, O, N atoms. All calculations were carried out using the Gaussian 09 software program (Wallingford, CT, USA) [10]. The molecular electrostatic potential (MEP) was obtained by the Gaussian 09 software program (Wallingford, CT, USA) [11]. 

## 3. Results and Discussion

### 3.1. Characterization of the Ag Nanoparticles (NPs)

Ag NPs are most commonly used SERS-active substrates in fundamental and applied sciences. The spheroidal Ag NPs used in this study have a maximum absorption around 430 nm with an average diameter of 60 nm, which are consistent with the results reported in the literature (Figure S1) [12]. 

### 3.2. Molecular Geometry

**Figure 1:**
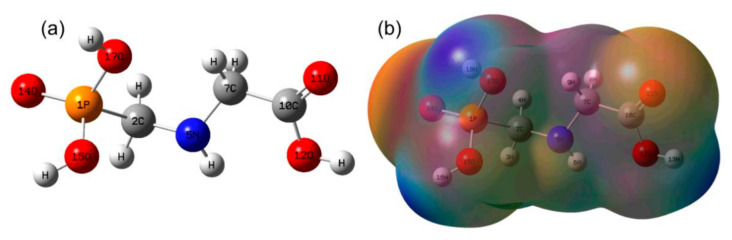
Optimized geometry and molecular electrostatic potential (MEP) mapping of glyphosate with/without 2‒^13^C‒isotope labeled (**a**) Optimized geometry of glyphosate with/without 2‒^13^C‒isotope labeled, (**b**) MEP mapping of glyphosate with/without 2‒^13^C‒isotope labeled.

The optimized geometry of 13–GLP or 12–GLP is shown in Figure 1a. In Figure 1a, ^13^C at C7 position. The corresponding structural parameters of bond lengths, bond angles, and dihedral angles are shown in Table 1. The atom numerical labels in the following discussion refer to Figure 1a. The MEP mapping of the 13–GLP or 12–GLP is presented in Figure 1b. MEP mapping provides a visual method to understand the relative polarity of a molecule. Notably, it is a very useful procedure to study the relationship between molecular structures and their physiochemical properties. In Figure 1b, negative charges (electrophilic regions) are represented in red, while positive charges (nucleophilic regions) are represented in green; the MEP increases in the order of red < orange < yellow < green < blue. The total electron density and MEP on the complex surfaces were generated at the B3LYP/6‒311++G(d,p) level of theory. 2‒^13^C‒glyphosate and glyphosate are only one isotopic atom apart, thus their structural parameters are exactly the same, as shown in Table S1 (Supplementary Materials). As we know, there is no difference in space configuration between isotope labeled molecules and protomolecules, and results in this report consistent with the results reported in the literature.

### 3.3. DFT Calculations of 13–GLP and 12–GLP

Each 13–GLP or 12–GLP molecule consists of 18 atoms, undergoes 48 normal modes of vibrations. The calculated vibration modes are listed in Table 1 and Table S2 (Supplementary Materials). Optimized geometry of 13–GLP and 12–GLP are the same, however, the vibrations calculated are different in some bonds.

**Figure 2:**
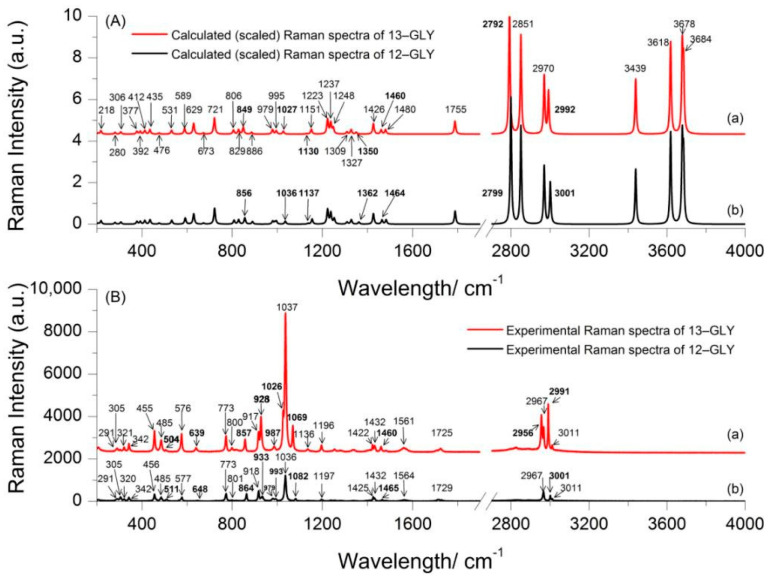
Experimental and calculated (scaled) Raman spectra of 2‒^13^C‒glyphosate (13–GLP) and glyphosate (12–GLP) in the spectral region 200 and 4000 cm^−1^ (**A**) calculated (scaled) Raman spectra of 13–GLP (a), calculated (scaled) Raman spectra of 12–GLP (b), (**B**) Experimental Raman spectra of 13–GLP (a), Experimental Raman spectra of 12–GLP (b).

The absence of imaginary frequencies in the DFT calculations indicates that the calculated 13–GLP and 12–GLP structures are minima on their respective potential energy surfaces. To fit the theoretical to the experimental wavenumbers, a scaling factor of 0.980 was employed to optimize the computed data under 1800 cm^−1^, and 0.9621 for wavenumbers higher than 1800 cm^−1^. The theoretical Raman spectra of the 13–GLP and 12–GLP calculated (scaled) at the B3LYP/6-311++G(d,p) level of theory is presented in Figure 2A(a,b), while the experimental Raman spectra in the solid state is presented in Figure 2B(a,b).

The calculated Raman modes of 13–GLP are observed at 412, 435, 476, 531, 589, 629, 673, 721, 806, 829, 849, 886, 978.6, 979.5, 995, 1027, 1130, 1151, 1223, 1237, 1248, 1309, 1327, 1350, 1426, 1460, 1480, 1755, 2792, 2851, 2970, 2992, 3439, 3618, 3678 and 3684 cm^−1^ (Table S2), Table 1 and Figure 2A(a). Results of the calculated frequencies of 12–GLP (Figure 2A(b)) were consistent with the results reported by Holanda et al. [13]. The frequencies of 13–GLP (Figure 2A(a)) in the DFT calculations indicate that most of the vibrational modes in calculated frequencies of 13–GLP agreed well with the theoretically predicted frequencies of 12–GLP. However, the isotope labeled atom changed the Raman intensity of some bonds and changed frequencies. Raman bands at 849, 1027, 1130, 1350 cm^−1^ for theoretical 13–GLP Raman spectrum appear at 856, 1036, 1137, 1362 cm^−1^ for theoretical 12–GLP assigned to C2N5C7C10 skeleton, C2N5C7 symmetric stretching, C2N5C7 asymmetric stretching, C7H_2_ wagging + C10C7 stretching, respectively. It is noteworthy that Raman band at 1426 and 1460 cm^−1^ of 13–GLP appear at 1426 and 1464 cm^−1^ of 12–GLP assigned to δ(CH_2_) from δ(C7H_2_) or δ(C2H_2_). Large shifts are also observed at wavenumbers higher than 1800 cm^−1^, Raman bands at 2792, 2992 cm^−1^ for theoretical 13–GLP Raman spectrum appear at 2799, 3001 cm^−1^ for theoretical 12–GLP assigned to C7H8 stretching, C7H9 stretching. However, C7H_2_ twisting has the same Raman spectrum appear at 1237 cm^−1^ in both 13–GLP and 12–GLP. As we know, in similar structures, the smaller the atomic mass of the chemical bond has a higher infrared absorption frequency. The isotopic substitution is expected to cause downshifts for those bands deriving from modes which involve the ^13^C atom. Thus, a downshift for band involves ^13^C atom isotopic substitution.

### 3.4. Experimental Raman Spectra of 13–GLP and 12–GLP

The observed vibration modes are listed in Table S2 (Supplementary Materials), with a tentative assignment, which is mainly based on previous work on glyphosate and related molecules [13,14,15,16]. Results of the frequencies of 12–GLP and 13–GLP (Figure 2B(a,b)) were consistent with the results reported [5]. The normal Raman modes of 13–GLP in the solid state are observed at 206, 291, 305, 320, 342, 456, 485, 511, 577, 639, 773, 800, 857, 917, 927, 987, 1026, 1036, 1069, 1136, 1197, 1255, 1283, 1338, 1425, 1432, 1460, 1482, 1564, 1729, 2956, 2967, 2991, and 3011 cm^−1^. Most of vibrational modes observed in the solid-state Raman spectrum of 13–GLP agreed well with frequencies of 12–GLP. Summary observed normal Raman scattering vibration modes of glyphosate 12–GLP and 13–GLP are listed in Table 1. However, the downshifts for some bands cause by ^13^C atom isotopic substitution can be assigned more accurately. As with the results of theoretical vibration, some Raman bands downshift were caused by the isotopic substitution. Raman bands at 504, 639, 928, 1069, 1350, and 1460 cm^−1^ for 13–GLP Raman spectrum in solid state appear at 511, 648, 933, 1082, and 1465 cm^−1^ for 12–GLP assigned to O12H bending and C7H_2_ bending, C2N5H twisting and C7N5H twisting, and C7H_2_ rocking, C7H_2_ bending. Calculated theoretical Raman spectrum of 12–GLP/13–GLP is different from experimental Raman spectrum, the relative intensities are not precisely predicted for all the Raman bands. However, it could show structural parameters of 12–GLP/13–GLP, as they are still fairly useful for the assignments of the normal modes in the Raman spectrum. Raman spectrum will be influenced by excitation wavelength, and this may explain these discrepancies. The shapes of theoretical spectrum can well conform to experiment, but the wavenumbers are underestimated by about 15 cm^−1^.

It is a remarkable fact that Raman shift of CH_2_ bending downshift from 1465 cm^−1^ for 12–GLP to 1460 cm^−1^ for 13–GLP, another Raman shift is still 1482 cm^−1^, however, in the calculated frequencies of CH_2_ bending, one Raman shift is 1426, another downshift from 1464 cm^−1^ to 1460 cm^−1^. 

Isotopic substitution (an atom is replaced by an isotope of larger mass) leads to a decrease in the wavenumber of all modes that involve the movements of the ^13^C atoms (harmonic oscillator: a reduces mass, μ, increases and vibrational energy level). According to the baseline of vibrational spectrum $\bar{\nu }=\frac{1}{2\pi c}\sqrt{\frac{k}{\mu }}$ shows that the larger the mass, the smaller the wavenumber. Therefore, isotopic substitution has opened the door to studying more complex systems. Thus, we assigned 1460 cm^−1^ for 13–GLP in normal Raman spectrum in solid state to C7H_2_ bending, and 1482 cm^−1^ to C2H_2_ bending. Similarly, we assigned 928 cm^−1^ for 13–GLP in normal Raman spectrum in solid state to C7H_2_ rocking.

### 3.5. Experimental FT–IR Spectra of 13–GLP and 12–GLP

**Figure 3:**
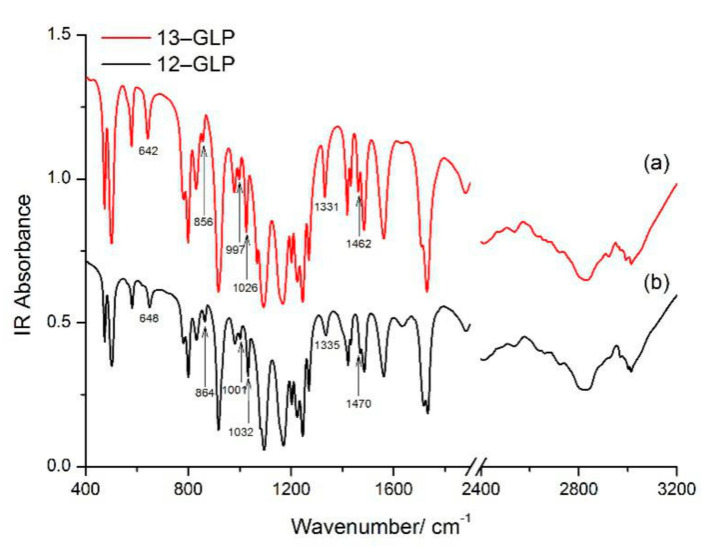
Experimental Fourier transform infrared (FT–IR) spectra of 2‒^13^C‒glyphosate (13–GLP) and glyphosate (12–GLP) in the spectral region 400 and 4000 cm^−1^ (**a**) IR spectra of 13–GLP, (**b**) FT–IR spectra of 12–GLP.

FT–IR spectrum and Raman spectrum can play complementary roles in vibration assignment. The observed vibration modes are listed in Table 1 and Table S2 (Supplementary Materials), with a tentative assignment. The normal IR modes of 13–GLP in the solid state are observed at 473, 501, 579, 642, 781 and 798, 829, 856, 916, 980, 997, 1026, 1067, 1094, 1167, 1202, 1223 and 1244, 1269, 1331, 1420, 1433, 1462, 1483, 1560, 1709 and 1730, 2411, 2536, 2833, 2922, 2991 cm^−1^. Most of vibrational modes observed in the solid-state IR spectrum of 13–GLP agreed well with frequencies of 12–GLP. However, the downshifts for some bands cause by ^13^C atom isotopic substitution can also be observed (Figure 3). IR bands at 642, 997, 1331, and 1462 cm^−1^ for 13–GLP in solid state appear at 648, 1001, 1335, and 1470 cm^−1^ for 12–GLP assigned to C2N5H twisting + C7N5H twisting, O15H wagging + O17H wagging, C10O12H bending+ C7H_2_ wagging, C7H_2_ bending. The downshifts for these bands cause by ^13^C atom isotopic substitution with mass changed.

The band at 864/857 cm^−1^ assigned to PO symmetric stretching and PC stretching also exist in Raman/IR shift with no bond to C7 atom. Feis et al. [5] consider (CO_2_)–(CNH_3_) as two-mass oscillator, downshift cause by mass changed. Likewise, the IR band at 1032/1026 cm^−1^ assigned to HOP bending also downshifted, yet only Raman shift of 13–GLP at 1026 cm^−1^ is observed, Raman shift of 12–GLP is not observed.

In conclusion, spectra shift by isotopic can be assigned vibrational modes more accurately because it does not change the symmetry of molecular structure and force constants between chemical bonds. The vibrational frequency of isotope substitution is shifted due to the difference of mass. In this paper, bands downshifts cause by ^13^C atom isotopic substitution to ^12^C.

### 3.6. Raman and SERS Spectra of PD POroduct from 12–GLP/13–GLP

Glyphosate Raman and SERS spectra have been subject of different experimental and theoretical studies, we also obtained SERS spectra of 13–GLP absorbed on Ag NPs. However, the observed SERS pattern in terms of a complex equilibrium between different orientation/conformation of the glyphosate molecules on the metal surface shows that sensitivity in SERS experiments is quite low [5]. 

**Figure 4:**
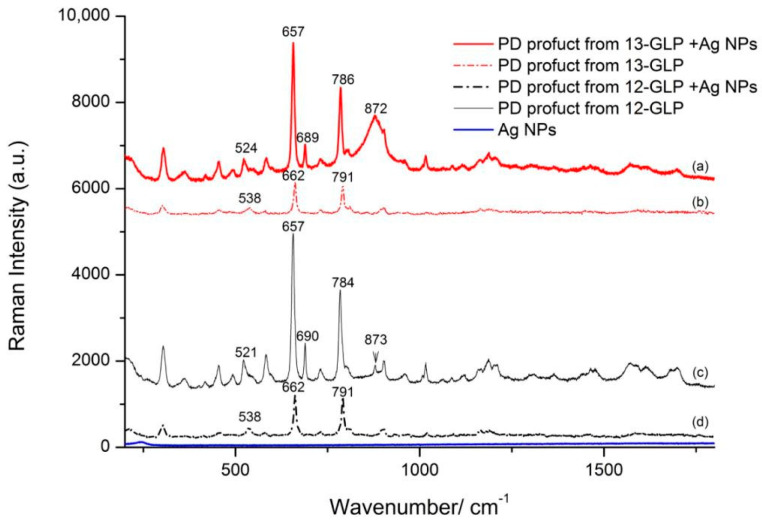
Raman and Surface-enhanced Raman scattering (SERS) spectra absorbed onto Ag NPs of the purple color dye (PD) products from glyphosate (12–GLP) and 2‒^13^C‒glyphosate (13–GLP) at a concentration of 1.0 × 10^−3^ mol·L^−1^. (**a**) SERS spectra of PD products from 12–GLP (**b**) Raman spectra of PD products from 12–GLP (**c**) SERS spectra of PD products from 13–GLP (**d**) Raman spectra of PD products from 13–GLP.

Pesticide molecules without SERS-active functional groups could be coupled by some probe molecules to achieve a better signal [17]. In previous work, we reported a glyphosate detection method based on ninhydrin reaction and surface−enhanced Raman scattering spectroscopy (SERS) to overcome this disadvantage. The PD product of the ninhydrin reaction is found to SERS-active and directly correlate with the glyphosate concentration. Meanwhile, experimental information about the structure changes of glyphosate and its PD product absorbed on Ag nanoparticles still needs further detection. Normal Raman and SERS spectra of PD product from 12–GLP/13–GLP with vibrational assignments are shown in Figure 4 and Table S3. They are clearly distinguishable with two relatively stronger peaks around 662 and 791 cm^−1^ in each spectroscopy. Figure 4a,c compares the Raman spectra of PD product from 13–GLP and 12–GLP are very similar, yet two band at 662 and 791 cm^−1^ display intensity changed due to mass changes caused by the ^13^C atom isotopic substitution. 

Furthermore, SERS spectra of PD product from 12–GLP/13–GLP are different when absorbed on Ag NPs. PD product from 12–GLP/13–GLP absorbed on Ag NPs, there are slight shifts of the two strong bands from 662 and 791 cm^−1^ in the normal Raman spectra to 657 and 784/786 cm^−1^, respectively. Raman intensity of the band at 662 and 791 cm^−1^ of normal PD product from 12–GLP/13–GLP are similar, however, SERS intensity are different, the peak at 662 cm^−1^ is much higher than at 784/786 cm^−1^. The band at 538 cm^−1^ shift to 521/524 cm^−1^ when absorbed on Ag NPs in SERS spectra. A strong peak appears at 690/689 cm^−1^ in SERS spectra that is not present in experimental Raman spectra (Figure 4). The peak at 872/873 cm^−1^ assigned to Mo–O from Na_2_MoO_4_ which is a catalyst forms a weak π-complex with aromatic rings of the ninhydrin component in the PD product. This peak in PD product from 13–GLP is much higher with wider full width of half maximum than from 12–GLP. 

This reaction is different from the classic amino acid color reaction with ninhydrin, in which no decarboxylation or dealdehyding reaction takes place. The molecular structure of PD product includes two moieties, N5 atom from glyphoate combines with ninhydrin, forming a new C−N bond and linking glyphoate and ninhydrin. Ninhydrin molecular is C_2_ symmetry, however, in PD product changed to C_1_ symmetry. The peaks at 657 and 786/784 cm^−1^ are assigned to the out–of–plane bending vibration of C=O from ninhydrin molecular. The peak 689 and 524/521 cm^−1^ are related to the vibration of the benzene ring carbon skeleton. This suggests that PD product interacts with the Ag atoms via the oxygen atoms of the ninhydrin moiety. According to the surface selection rule, we infer the PD product molecules adsorbed on the surface had a perpendicular orientation. ^13^C atom isotopic substitution to ^12^C change the mass, thus PD product coupled MoO_4_^2−^ with a weak π-complex slightly changes the adsorption orientation on Ag NPs. 

Glyphosate Raman and SERS spectra have been the subject of different experimental and theoretical studies but sensitivity in SERS experiments is quite low due to small Raman cross-section and complex equilibrium between different orientation/conformation of the adsorbed molecules on the metal surface. –COO− group does not participate in the interaction of the glyphosate with Ag NPs. Imidazole ring in PD product interacts with the Ag NPs surface, adopting almost erect state orientation relative to it. None of these bands can be attributed to the vibrations of glyphosate, therefore these moieties do not affect the SERS spectrum of glyphosate on the surface of Ag NPs. Thus, the combination of high Raman scattering probe with isotopic labeling provides avenues for accurate and quantitative SERS. We believe such a method will open a new promising area for isotopic SERS to investigate the mechanism of surface photoinduced reactions through tracking the fingerprint information changes of the species on metal nanostructures.

### 3.7. Quantitative Analysis of 13–GLP

**Figure 5:**
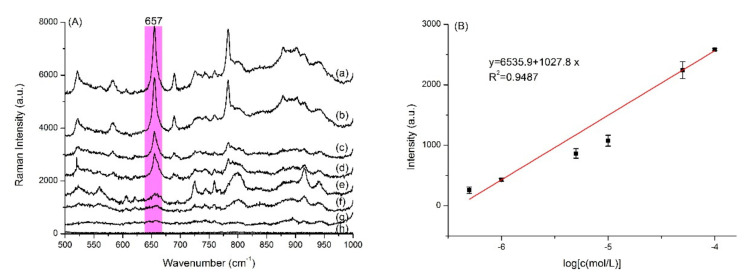
(**A**) Representative concentration-depend SERS spectra of (a) 1.0 × 10^−4^, (b) 5.0 × 10^−5^, (c) 1.0 × 10^−5^, (d) 5.0 × 10^−6^, (e) 1.0 × 10^−6^ mol·L^−1^, (f) 5.0 × 10^−7^ mol·L^−1^, (g) 1.0 × 10^−7^ mol·L^−1^ purple color dye product from 2‒^13^C‒glyphosate (13–GLP) and (h) blank; the standard curve (**B**). Excitation wavelength: 633 nm.

We examined the SERS spectra of the PD products from 13–GLP at different concentrations shown in Figure 5. The SERS intensity at 657 cm^−1^ shows a good linear relation with the concentrations of PD product in the range of 5.0 × 10^−7^−1.0 × 10^−4^ mol·L^−1^ (*y* = 6535.9 + 1027.8 × lgC, R^2^ = 0.9487). The limit of detection (LOD) is thus calculated to be 4.3 × 10^−7^ mol·L^−1^. Limit of quantitation (LOQ) is 5.0 × 10^−7^ mol·L^−1^. Recoveries for tap water (1.0 × 10^−6^ mol·L^−1^, 5.0 × 10^−5^ mol·L^−1^, 1.0 × 10^−5^ mol·L^−1^) were 83.8–115.5%, relative standard deviation (RSD) were 1.1‒16.8%.

## 4. Conclusions

Glyphosate Raman and SERS spectra have been the subject of different experimental and theoretical studies, however, sensitivity in SERS experiments is still quite low due to the complex equilibrium between different orientation/conformation of the adsorbed molecules on the metal surface. 2‒^13^C isotopically substituted derivative is used to confirm the vibration modes in Raman and FT‒IR spectra. The isotopic substitution is expected to cause downshifts deriving from modes which involve the ^13^C atom, and PO symmetric stretching and PC stretching with no bond to ^13^C atom. This downshift caused by isotopic substitution is also present in DFT calculations, though there is no difference in space configuration between isotope labeled molecules and protomolecules. We further reported the SERS spectra of 13‒GLP by combining ninhydrin reaction on Ag NPs coupled by probe molecules and thus bind to the substrates. This indirectly offers the opportunity to promote interactions with Ag NPs and reduce the complex equilibrium between different orientation/conformation of glyphosate molecules on the metal surface with a LOD of 4.38 × 10^−7^ mol·L^−1^ and LOQ of 5.0 × 10^−7^ mol·L^−1^. 2‒^13^C isotopically substituted derivative is used to confirm the vibration modes in Raman and FT‒IR spectra more conveniently with higher sensitivity, better reproducibility, and lower relative standard deviation. This work will open a new promising area for isotopic SERS to investigate the mechanism of surface photoinduced reactions through tracking the fingerprint information changes of the species on metal nanostructures.

## Figures and Tables

**Table 1:** Summary calculated and observed normal Raman scattering/Fourier Transform Infrared vibration modes of glyphosate (12–GLP) and 2‒^13^C‒glyphosate (13–GLP).

Theoretical Vibrations Frequency (After Scaling)		Experimental Raman (Solid State)		Experimental IR (Solid State)		Vibrational Assignments
12–GLP	13–GLP	12–GLP	13–GLP	12–GLP	13–GLP	
532	531	511	504	501	501	δ(O12H) + δ(C7H_2_)
629	629	648	639	648	642	τ(C2N5H) + τ(C7N5H)
674	673	‒	‒	‒	‒	ω(C10O12H)
829	829	864	857	864	856	νs(PO) + ν(PC)
856	849	918	917	916	916	C2N5C7C10 skel.
890	886	933	928	‒	‒	ρ(C7H_2_)
979	979	979	‒	982	980	ρ(C2H_2_)
989	979.5	993	987	1001	997	ω(O15H) + ω(O17H)
996	995	‒	1026	1032	1026	δ(HOP)
1036	1027	1037	1036	1082	1067	νs(C2N6C7)
1137	1130	1082	1069	1095	1094	νas(C2N5C7)
1310	1309	1340	1338	1335	1331	δ(C10O12H) + ω(C7H_2_)
1328	1327	1422	1425	1421	1420	ω(C7H_2_)
1362	1350	1432	1432	1433	1433	ω(C2H_2_) + ν(C10C7)
1426	1426	1465	1460	1470	1462	δ(C7H_2_)
1464	1460	1482	1482	1485	1483	ω(NH_2_) + δ(C2H_2_)
2799	2792	‒	‒	2409	2411	ν(C7H8)
3001	2992	3001	2991	3001	2922	ν(C7H9)

s: symmetry; as: asymmetry; ν: stretching vibration; δ: bending; ρ: rocking; ω: wagging; τ for twisting; skel: skeleton.

## Supplementary Materials

The following are available online at https://www.mdpi.com/2079-4991/10/12/2539/s1, Figure S1: UV-Vis spectrum of the Ag NPs, Table S1: The optimized structural parameters of 2‒13C‒glyphosate or glyphosate calculated at the B3LYP/6-311+G** level, Table S2: Calculated and normal Raman spectra of glyphosate (12‒GLY) and 2‒13C‒glyphosate (13‒GLY) in the frequencies, Table S3: Raman and SERS band assignments (cm−1) of the Purple Color Dye (PD) product from glyphosate (12–GLP) and 2‒13C‒glyphosate (13–GLP).
