# Supplementary material for: Role of 2‒13C Isotopic Glyphosate Adsorption on Silver Nanoparticles Based on Ninhydrin Reaction: A Study Based on Surface—Enhanced Raman Spectroscopy
Source: Nanomaterials (Basel). 2020 Dec 17;10(12):2539. doi: 10.3390/nano10122539 (PMC7766329; doi:10.3390/nano10122539)
Supplement: Supplementary file 1 [file nanomaterials-10-02539-s001.pdf]

Supplementary Materials: Role of 2–^{13}C Isotopic Glyphosate Adsorption on Silver Nanoparticles Based on Ninhydrin Reaction: A Study Based on Surface—Enhanced Raman Spectroscopy

Meng-Lei Xu, Yu Gao, Jing Jin, Jin-Feng Xiong, Xiao Xia Han and Bing Zhao

Table S1. The optimized structural parameters of 2–^{13}C–glyphosate or glyphosate calculated at the B3LYP/6-311+G** level.

BL	Value (Å)	BA	Value (°)	Dihedral	Value (°)
P1–C2	1.831	C2–P1–O14	115.0698	O14–P1–C2–H3	−59.242
P1–O14	1.4833	C2–P1–O15	106.7133	O14–P1–C2–H4	53.2311
P1–O15	1.6268	C2–P1–O17	101.1582	O14–P1–C2–N5	−179.3571
P1–O17	1.6207	O14–P1–O15	112.8636	O15–P1–C2–H3	66.7966
C2–H3	1.0928	O14–P1–O17	116.7861	O15–P1–C2–H4	179.2697
C2–H4	1.1019	O15–P1–O17	102.7792	O15–P1–C2–N5	−53.3185
C2–N5	1.4524	P1–C2–H3	104.2248	O17–P1–C2–H3	173.9318
N5–H6	1.0113	P1–C2–H4	106.0652	O17–P1–C2–H4	−73.595
N5–C7	1.4563	P1–C2–N5	115.1935	O17–P1–C2–N5	53.8167
C7–H8	1.1069	H3–C2–H4	106.7418	C2–P1–O15–H16	−116.518
C7–H9	1.0894	H3–C2–N5	109.6024	O14–P1–O15–H16	10.8367
C7–C10	1.5133	H4–C2–N5	114.235	O17–P1–O15–H16	137.5027
C10–O11	1.203	C2–N5–H6	111.1038	C2–P1–O17–H18	175.0585
C10–O12	1.3571	C2–N5–C7	115.2744	O14–P1–O17–H18	49.3701
O12–H13	0.9691	H6–N5–C7	110.8593	O15–P1–O17–H18	−74.741
O15–H16	0.9652	N5–C7–H8	112.965	P1–C2–N5–H6	143.783
O17–H18	0.9655	N5–C7–H9	110.2961	P1–C2–N5–C7	−89.0384
		N5–C7–C10	113.1169	H3–C2–N5–H6	26.6667
		H8–C7–H9	106.8483	H3–C2–N5–C7	153.8453
		H8–C7–C10	105.8793	H4–C2–N5–H6	−93.0458
		H9–C7–C10	107.3522	H4–C2–N5–C7	34.1328
		C7–C10–O11	124.8115	C2–N5–C7–H8	−51.7492
		C7–C10–O12	112.385	C2–N5–C7–H9	67.7455
		O11–C10–O12	122.7406	C2–N5–C7–C10	−172.0143

C10–O12–H13	107.4151	H6–N5–C7–H8	75.5527
P1–O15–H16	110.8703	H6–N5–C7–H9	−164.9525
P1–O17–H18	114.1316	H6–N5–C7–C10	−44.7124
		N5–C7–C10–O11	−151.7221
		N5–C7–C10–O12	31.1034
		H8–C7–C10–O11	84.0493
		H8–C7–C10–O12	−93.1253
		H9–C7–C10–O11	−29.8163
		H9–C7–C10–O12	153.0091
		C7–C10–O12–H13	178.728
		O11–C10–O12–H13	1.4859

Table S2. Calculated and normal Raman spectra of glyphosate (12–GLY) and 2–^{13}C–glyphosate (13–GLY) in the frequencies

Theoretical Raman		Theoretical Raman (after scaling)		Experimental Raman (solid state)		Experimental IR (solid state)		Vibrational assignments			
12–GLP	13–GLP	12–GLP	13–GLP	12–GLP	13–GLP	12–GLP	13–GLP	From [1]	From [3]	From [4]	Assignments
32	32	31	31	–	–	–	–			γ(C10C7N5C2) + γ(O12C10C7N5) + γ(O17PC2N5) + γ(C7N5C2P)	γ(C10C7N5C2) + γ(O12C10C7N5) + γ(O17PC2N5) + γ(C7N5C2P)
44	44	43	43	–	–	–	–			γ(C7N5C2P) + γ(O17PC2N5) + γ(C10C7N5C2) + γ(O12C10C7N5)	γ(C7N5C2P) + γ(O17PC2N5) + γ(C10C7N5C2) + γ(O12C10C7N5)
58	59	57	58	–	–	–	–			δ(PC2N5) + (C7N5C2) + δ(C10C7N5)	δ(PC2N5) + δ(C10C7N5)
112	111	110	109	–	–	–	–			γ(HOPC) + δ(O17PO14) + γout (O11C5O17P)	γ(HO17PC) + δ(O17PO14) + γout(O11C5O17P)
128	128	126	126	–	–	–	–			γ(O12C10C7N1) + γ(C10C7N5C2) + γ(C7N5C2P)	γ(O12C10C7N1) + γ(C10C7N5C2) + γ(C7N5C2P)
172	171	168	167	–	–	–	–			γ(O17PC2N5) + γ(C7N5C2P) + γ(C10C7N5C2)	γ(O17PC2N5) + γ(C7N5C2P) + γ(C10C7N5C2)

210	209	206	205	206	206	–	–			δ(C10C7N5) + γout(O14C2O15P) + δ(O12C10C17) + δ(PC2N5)	δ(C10C7N5) + δ(O12C10C17)
223	223	218	218	–	–	–	–			δ(O15PO17) + γ(O17PC2H) + ν(P1C2) + δ(C7N5C2)	γ(O17PC2H) + δ(C7N5C2)
286	286	280	280	291	291	–	–	skeletal motion [2]		γ(HO17PC2) + δ(O17PO14)	γ(HO17PC2) + δ(O17PO14)
312	312	306	306	303	305	–	–			γ(HOPC) + δ(O15PO14)	γ(HOPC) + δ(O15PO14)
385	385	377	377	321	320	–	–			γout(O15C2O17P) + δ(O12C10C7) + δ(O14PO15)	γout(O15C2O17P) + δ(O14PO15)
400	400	392	392	342	342	–	–	τCH_2 [2]		δ(O17PO14) + γout(OCOP)	δ(O14PO17) + ϱ(C2H_2)
421	421	412	412	–	–	–	–			δ(O10PO11) + γout(O10C2O15CP)	δ(O10PO11)
444	444	436	435	456	455	–	–	δ(PO_3) + δ(NCCO)	ϱ(CH_2) + δ(OH)	δ(OPO) + δ(O12C7C10)	δ(OPO) + δ(O12C7C10)
486	485	476	476	485	485	473	473	δ(HOPO) + ϱ(PCN) + δ(NCC) + δ(HOCO) + ϱ(CH_2)	δ(OH) + ϱ(CH_2) + (PO_2)	γ(OCOP) + δ(O12C10C7) + γ(N5C7C10H) + δ(P1C2N5)	δ(OH) + ϱ(C7H_2) + ω(PO_2)
543	542	532	531	511	504	501	501	ϱ(HOPO) + δ(CNC) + δ(HOCO) + ϱ(CH_2)	δ(OH) +δ(CH)	γout(O12C7C10H) + γout(O11C7O11C10) + δ(O11C10O12)	δ(O12H) + δ(C7H_2)
604	601	592	589	576	577	581	579	δϱ(PO_3) + skel(NCCOO)	δ(OH) + δ(OH–C=O)	δ(O11C10O12) + γout(O12C7C10H)	δ(OH) + δ(OH–C=O)

642	642	629	629	648	639	648	642	ν(PC) + δ(NCC) + δ(COO)		γout(O15C7O12C10) + γ(O12C10C7H) + δ(O12C10O11)	τ(C2N5H) + τ(C7N5H)
687	687	674	673	–	–	–	–			ν(PC2) + ν(PO15)	ω(C10O12H)
737	736	722	721	773	773	781 and 798	781 and 798	ν(PC) [1]	δ(NH) + ϱ(CH2) + ν(P–OH)	γout(N5C7C10H) + δ(N5C7H)	ω(C2N5H)
825	822	808	806	801	800	831	829	ν(PC) + ϱ(CH2) + ϱ(NH2) + ν(CCOO)		ν(PO) + ν(P1C2)	ν(PO15)
846	846	829	829	864	857	864	856	ν(C–C)	ϱ(CH_2) + δ(NH) + ν(C–C)	ν(PO)	νs(PO) + ν(PC)
874	867	856	849	918	917	916	916	CNCC skel.	ϱ(CH_2)	ν(C10C7) + ν(C2H_2)	CNCC skel.
908	904	890	886	933	928	–	-	νs(PO_3) + ν(PC)		ν(C10C7) + ν(C2H_2) + γout(C7C10O12H)	ϱ(C7H_2)
999	999	979	979	979	–	982	980		ϱ(CH_2) + δ(OH)	δ(HOP)	ϱ(C2H_2)
1009	1000	989	980	993	987	1001	997	νs(PO_3) + τ(CH2) + ϱ(NH_2) + CNCC skel.		τ(C7H_2) + γout(C7C10O12)	ω(O15H) + ω(O17H)
1016	1016	996	995	–	1026	1032	1026			δ(HOP)	δ(HOP)
1057	1048	1036	1027	1037	1036	1082	1067		νs(PO_2) + δ(OH)	ν(NC)	νs(C2N6C7)
1160	1153	1137	1130	1082	1069	1095	1094	νa(PO_3) + ν(C–N)	ν(C–N) + ν(C–OH)	ν(O12C10) + δ(C10O12H) + ν(N5C2)	νas(C2N6C7)

1178	1175	1154	1151	1135	1136	1171	1167	νa(POH)		ν(NC)	ν(O12C10) + δ(C10O12H) + ν(N5C2)
1248	1248	1223	1223	1196	1197	1203	1202	δ(CH_2 + NH_2 + CH_2) + ν(COH) + ν(CN)	τ(CH_2) + δ(OH)	τ(CH2)	ν(COH)
1263	1263	1237	1237	1253	1255	1223 and 1246	1223 and 1244	ν(PC) + ν(POH) + ω(CH_2)	τ(CH_2)	τ(CH2)	τ(C7H_2)
1278	1274	1253	1248	1280	1283	1269	1269	ω(CH_2) + ωτ(CH_2) + δ(COH) + ν(PC)	ω(CH_2)	τ(CH2)	τ(C2H_2)
1336	1336	1310	1309	1340	1338	1335	1331	ωτ(CH_2) + δ(COH) + δ(CNC) / ν(POH) + δ(CH_2) + ν(CCO)	ω(CH_2) + ν(C–C)	δ(O12H) + ω(C7H_2)	δ(C10O12H) + ω(C7H_2)
1355	1355	1328	1327	1422	1425	1421	1420	δ(C2H_2) + δ(POH)	δ(CH_2)	ω(C2H_2)	ω(C7H_2)
1390	1378	1362	1350	1432	1432	1433	1433	δ(C7H_2) + δ(POH)	δ(CH_2)	ω(C7H_2) + ν(O15C10) + ν(C10C7)	ω(C2H_2) + ν(C10C7)
1455	1455	1426	1426	1465	1460	1470	1462	ν(CC) + δ(CNH) / τ(NH_2)		δ(CH_2)	δ(C7H_2)
1494	1490	1464	1460	1482	1482	1485	1483	ω(NH_2) + δ(POH)		δ(CH_2)	ω(NH_2) + δ(C2H_2)
1513	1510	1482	1480	1561	1564	1560	1560	δ(NH_2)		δ(N5H)	δ(NH_2)
1825	1824	1756	1755	1725	1729	1717 and 1732	1709 and 1730	ν(C=O)	ν(C=O)	ν(C=O)	ν(C=O)
2910	2902	2799	2792	–	–	2409	2411			νas(CH_3)	ν(C7H8)

2963	2963	2851	2851	–	2956	2536	2536	$\nu s(C2H_2)$	ν(C2H4)
3087	3087	2970	2970	2967	2967	2827	2833	$\nu as(C7H_2)$	ν(C2H9)
3119	3110	3001	2992	3001	2991	3001	2922	$\nu as(C2H_2)$	ν(C7H9)
3575	3575	3439	3439	3011	3011	3013	2991	ν(N5H)	ν(N5H)
3761	3761	3618	3618	–	–	–	–	ν(O11H)	ν(O12H)
3823	3823	3678	3678	–	–	–	–	ν(OH)	ν(O17H)
3830	3830	3684	3684	–	–	–	–	ν(OH)	ν(O15H)

δ: bending; ν: stretching vibration; s: symmetry; as: asymmetry; β: bending; ϱ: rocking; ω: wagging; τ for twisting; skel: skeleton; γ: torsion.

Table S3. Raman and SERS band assignments (cm−1) of the Purple Color Dye (PD) product from glyphosate (12–GLP) and 2–13C–glyphosate (13–GLP)

Experimental Raman		Experimental SERS		Vibrational assignments [a,b]
12–GLP	13–GLP	12–GLP	13–GLP	
303	303	303	305	β(PO_2)
360	361	360	358	δ(O14PO17) + ρ(C2H_2)
402	403	403	401	δ(OPO) + δ(O12C7C10)
414	413	417	422	δ(O10PO11)
456	455	454	455	γ(CH_2) + β(OH)
494	492	492	493	β(OH) + γ(CH_2) + ϱ(PO_2)
538	538	521	524	β(OH) + β(CH)
578	577	583	582	δ(OH) + δ(OH–C=O)
662	662	657	657	o.p.β(C=O)
–	–	690	689	9a + ring5 breathing
731	731	730	730	ω(C2N5H)
792	791	784	786	o.p.β(C=O)
810	811	800	806	γ(CH2) + ν(C–C)
–	–	880	872	ν(Mo–O)
904	902	904	902	1 + a.v.(C–C)
–	–	961	–	19b + a.s.β(C=O)
1009	1010	1009	1008	ω(O15H) + ω(O17H)
1016	1020	1017	1016	19b + a.s.β(C=O) + β(O–C–C)
1065	1066	1064	1071	δ(HOP)
1187	1086	1087	1085	ν(C–N) + β(C–OH)
1120	1120	1120	1112	τ(CH_2) + β(OH)
1164	1166	1166	1167	β(O–C–O)
1187	1188	1183	1186	19a + β(O–H)
1202	1202	1210	1209	3
1262	1262	1263	1262	β(O–H)
1311	1312	1312	1309	15
1367	1365	1365	1364	18a

1453	1452	1442	1446	β(CH_2)
–	–	1475	–	β(CH_2)
–	–	1570	1569	9a
1585	1583	1588	1589	9b

[a] The Wilson notation is employed. [b] s: symmetry; as: asymmetry; ν: stretching vibration; β: bending; γ: rocking; ω: wagging; i.p.: in-plane; o.p.: out-of-plane.

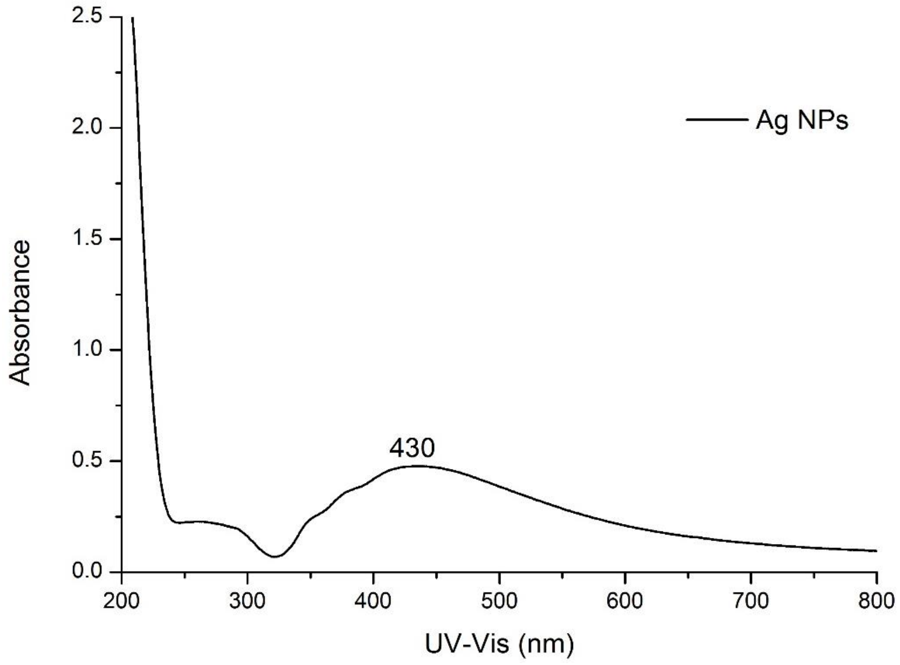

Figure S1. UV-Vis spectrum of the Ag NPs

Reference

1. Yael, J.A.; Fuhr, J.D.; Bocan, G.A.; Millone, A.D.; Tognalli, N.; Afonso, M.S.; Martiarena, M.L. Abiotic Degradation of glyphosate into aminomethylphosphonic acid in the presence of metals. *J. Agric. Food Chem* **2014**, *62*, 9651–9656.

2. Parameswari, A.; Asath, R.M.; Premkumar, R.; Benial, A.M.F. SERS and quantum chemical studies on N-methylglycine molecule on silver nanoparticles. *J. Mol. Struct* **2017**, *1138*, 102–109.

3. Costa, J.C.S.; Ando, R.A.; Sant'Ana, A.C.; Corio, P. Surface-enhanced Raman spectroscopy studies of organophosphorous model molecules and pesticides. *Phys. Chem. Chem. Phys* **2012**, *14*, 15645–15651.

4. Holanda, R.O.; Silva, C.B.; Vasconcelos D.L.M.; Freire, P.T.C. High pressure Raman spectra and DFT calculation of glyphosate. *Spectrochim. Acta A* **2020**, *242*, 118745–118753.
